# Blockade of inhibitory killer cell immunoglobulin-like receptors and IL-2 triggering reverses the functional hypoactivity of tumor-derived NK-cells in glioblastomas

**DOI:** 10.1038/s41598-022-10680-4

**Published:** 2022-04-26

**Authors:** Cüneyt Sönmez, Johannes Wölfer, Markus Holling, Benjamin Brokinkel, Walter Stummer, Heinz Wiendl, Christian Thomas, Andreas Schulte-Mecklenbeck, Oliver M. Grauer

**Affiliations:** 1 Department of Neurology With Institute of Translational Neurology, University Hospital Münster, Albert-Schweitzer-Campus 1, Building A1, 48149 Münster, Germany; 2 Department of Neurosurgery, University Hospital Münster, Münster, Germany; 3 Institute of Neuropathology, University Hospital Münster, Münster, Germany; 4 Present Address: Department of Spine Surgery, Klinikum Herford, 32049 Herford, Germany; 5 Present Address: Department of Neurosurgery and Spine Surgery, Hufeland Klinikum GmbH, 99974 Mühlhausen, Germany

**Keywords:** CNS cancer, Cancer microenvironment

## Abstract

Killer cell immunoglobulin-like receptors (KIRs) comprise a group of highly polymorphic inhibitory receptors which are specific for classical HLA class-I molecules. Peripheral blood and freshly prepared tumor cell suspensions (n = 60) as well as control samples (n = 32) were investigated for the distribution, phenotype, and functional relevance of CD158ab/KIR2DL1,-2/3 expressing NK-cells in glioblastoma (GBM) patients. We found that GBM were scarcely infiltrated by NK-cells that preferentially expressed CD158ab/KIR2DL1,-2/3 as inhibitory receptors, displayed reduced levels of the activating receptors CD335/NKp46, CD226/DNAM-1, CD159c/NKG2C, and showed diminished capacity to produce IFN-γ and perforin. Functional hypoactivity of GBM-derived NK-cells persisted despite IL-2 preactivation. Blockade with a specific KIR2DL-1,2/3 monoclonal antibody reversed NK-cell inhibition and significantly enhanced degranulation and IFN-γ production of IL-2 preactivated NK-cells in the presence of primary GBM cells and HLA-C expressing but not HLA class-I deficient K562 cells. Additional analysis revealed that significant amounts of IL-2 could be produced by tumor-derived CD4^+^ and CD8^+^CD45RA^-^ memory T-cells after combined anti-CD3/anti-CD28 stimulation. Our data indicate that both blockade of inhibitory KIR and IL-2 triggering of tumor-derived NK-cells are necessary to enhance NK-cell responsiveness in GBM.

## Introduction

Natural killer cells (NK-cells) play a critical role in host defense and tumor surveillance by secreting immunostimulatory cytokines and by the killing of infected or transformed cancer cells^1^.

Two major NK-cell subsets can be discriminated based on the density of CD56 expression. CD56^dim^ NK-cells represent about 90% in peripheral blood, whereas CD56^bright^ NK-cells are more frequent in tissues and secondary lymphoid organs. CD56^dim^ cells display low proliferative capacity, express high levels of cytolytic granules containing perforin and granzymes and release proinflammatory cytokines such as IFN-γ after proper NK-cell triggering. CD56^bright^ cells proliferate efficiently in response to IL-2 or IL-15, are scarcely cytolytic but are potent cytokine producers early after NK-cell activation^2,3^.

Triggering of NK effector functions is regulated by several activating and inhibitory receptors which recognize a variety of ligands expressed on potential target cells. Human NK-cells express two different classes of inhibitory receptors: the highly polymorphic killer cell immunoglobulin-like receptors (KIRs) which survey potential target cells for the expression of specific epitopes presented on human leucocyte antigen class I molecules (HLA-A,B,C), and the CD94/NKG2A receptors which are specific for the highly conserved and ubiquitously expressed non-classical HLA-E molecules. During NK-cell differentiation, CD94/NKG2A is mainly expressed on more immature CD56^bright^ NK cells, whereas KIR expression increases with CD56^dim^ NK-cell maturation^4,5^.

KIRs are defined by the number of extracellular immunoglobulin-like domains (D) and the length of the intracytoplasmic tail. Inhibitory KIRs carry long cytoplasmic tails (L) containing immunoreceptor tyrosine-based inhibitory motifs (ITIM) that transduce inhibitory signals to NK-cells, whereas activating KIRs have short cytoplasmic tails (S). Inhibitory KIRs with two (KIR2DL) or three (KIR3DL) extracellular Ig-domains are specific for HLA-C or HLA-A/B allotypes, respectively. KIR2DL-1 binds HLA-Cw2, HLA-Cw4, HLA-Cw5 and HLA-Cw6 (called HLA-C2 group alleles), whereas KIR2DL-2 and KIR2DL-3 bind to HLA-Cw1, HLACw3, HLA-Cw7 and HLA-Cw8 (called HLA-C1 group alleles). Collectively, the inhibitory KIR2DL-1, -2, and -3 receptors recognize essentially all HLA-C allotypes^6–9^.

Therapeutic monoclonal antibodies (mAbs) directed against common inhibitory KIR2DL-1 and KIR2DL-2/3 have been tested in vitro and in early clinical trials. IPH2101 (formerly, 1-7F9), a recombinant fully human IgG4 mAb was shown to enhance NK-cell cytokine production and NK-cell mediated lysis of autologous leukemia (AML) blasts and multiple myeloma cells but not normal autologous cells^10–12^. Moreover, IPH2101 synergized with lenalidomide to increase NK cytotoxicity in myeloma patients^13,14^. Combinations with anti-CD20 mAbs have been successfully evaluated in patients with lymphoma^15^. Lirilumab, a second generation and hinge-stabilized fully human IgG4 mAb (BMS-986015, formerly IPH2102) was further investigated in patients with solid tumors and hematologic malignancies and with and without azacytidine in patients with myelodysplastic syndromes^16,17^.

In the present study, we aimed to analyze the frequency, phenotype, and function of KIR2DL-1,-2/3 expressing NK-cells in glioblastoma patients and to evaluate whether targeting of KIR2DL-1,-2/3 augments the cytolytic activity of tumor-derived NK-cells. We found that both IL-2 triggering and blockade of inhibitory KIRs are necessary to break the functional hypoactivity of GBM-derived NK-cells.

## Results

### GBM are infiltrated by low numbers of CD3-CD56^+^ NK-cells with preferential expression of KIR2DL-1,-2/3

**Figure 1:**
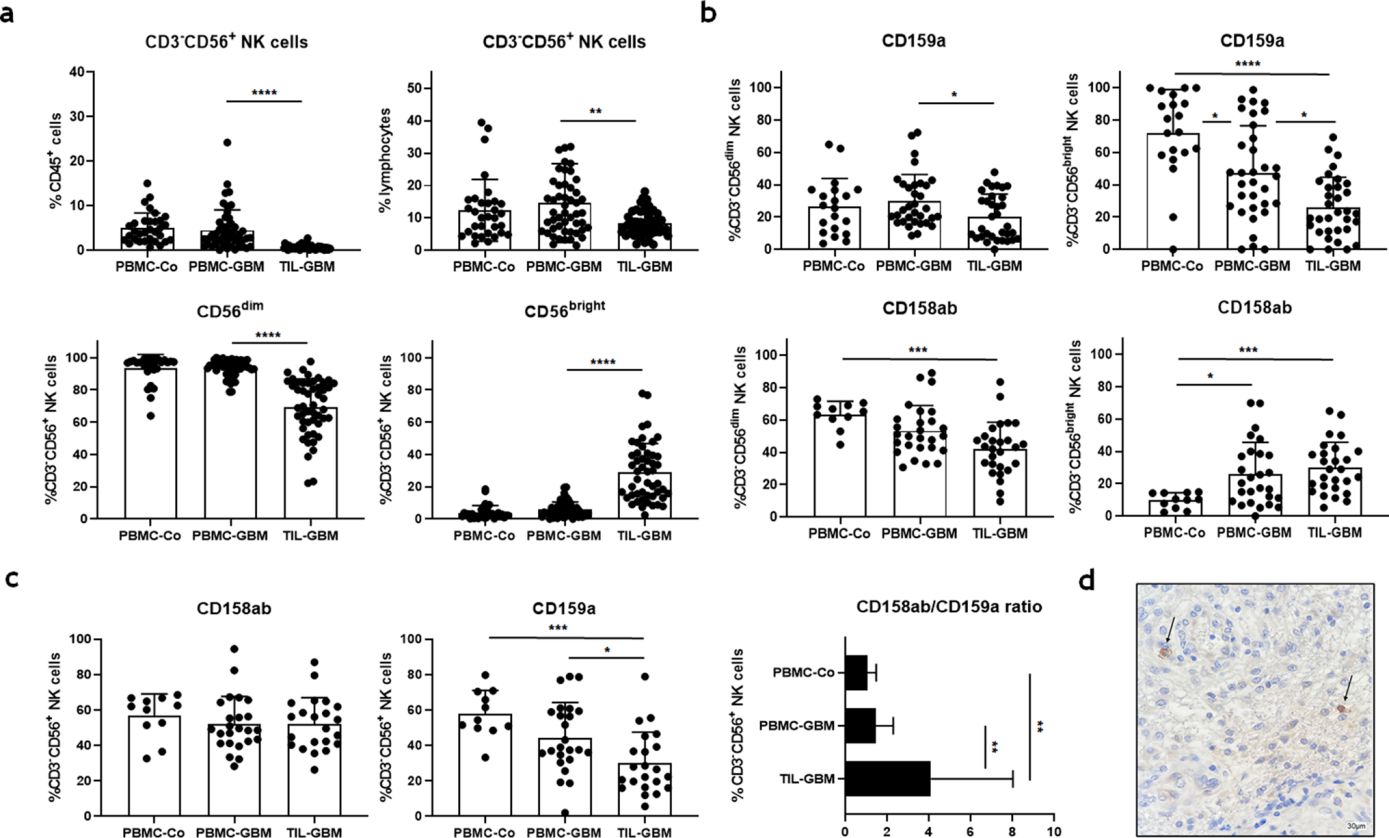
Frequency, phenotype, and distribution of NK-cells within peripheral blood and tumor cell suspensions of GBM patients opposed to control individuals. (**a**) The frequencies of CD3^-^CD56^+^ NK-cells within the CD45^+^ leucocyte and lymphocyte population, as well as the proportion of CD56^dim^ and CD56^bright^ NK-cells were calculated (**b**). The frequencies of CD159a/NKG2A^+^ and CD158ab/KIR2DL-1,-2/3 in CD56^dim^ and CD56^bright^ NK-cell subsets in peripheral blood (PBMC-GBM) and tumor cell suspensions of GBM patients (TIL-GBM) and control individuals (PBMC-Co) are shown. (**c**) In addition, the proportion of CD158ab^+^ and CD159a^+^ tumor-derived CD3^-^CD56^+^ NK-cells, as well as the ratio of CD158ab to CD159a compared to peripheral blood of GBM patients or control individuals were determined. (**d**) Representative immunohistochemical staining of KIR2DL-1/-2/3-expressing cells with glioblastoma tissue. Mean values + /− SD are depicted. P values were calculated using the Kruskal–Wallis test with post-hoc Dunn´s multiple comparison analysis (*p < 0.05, **p < 0.01, ***p < 0.001, ****p < 0.0001).

We found that CD3^-^CD56^+^ NK-cells comprised only a minor fraction of CD45^+^ leucocytes in tumor tissue when compared to peripheral blood of GBM patients. Similar results were obtained by calculating the percentage of NK-cells within the lymphocyte population in both compartments. The bulk of blood NK-cells were CD56^dim^ cells, whereas CD56^bright^ cells were found at low frequencies. In contrast, the percentage of CD56^dim^ cells was significantly reduced within the TIL population, while the relative frequency of CD56^bright^ cells was increased (Fig. 1a). Further analysis revealed significantly reduced percentages of CD159a/NKG2A^+^ CD56^dim^ and CD56^bright^NK-cells within tumor tissue when compared to peripheral blood of GBM patients, whereas the percentages of CD158a/KIR2DL-1^+^ and CD158b/KIR2DL-2/3^+^ CD56^dim^ and CD56^bright^ NK-cells were not different (Fig. 1b). Comparing the numbers of CD158ab and CD159a revealed a significantly higher ratio expressed by NK-cells isolated from tumor cell suspensions as compared to peripheral blood of GBM patients or control individuals (Fig. 1c). Immunohistochemistry confirmed scarce infiltration of CD158ab/KIR2DL-1,-2/3-expressing cells in GBM tissue (Fig. 1d).

### Impaired expression of activating receptors and functional hypoactivity of KIR2DL-1,-2/3^+^ GBM-derived NK-cells

**Figure 2:**
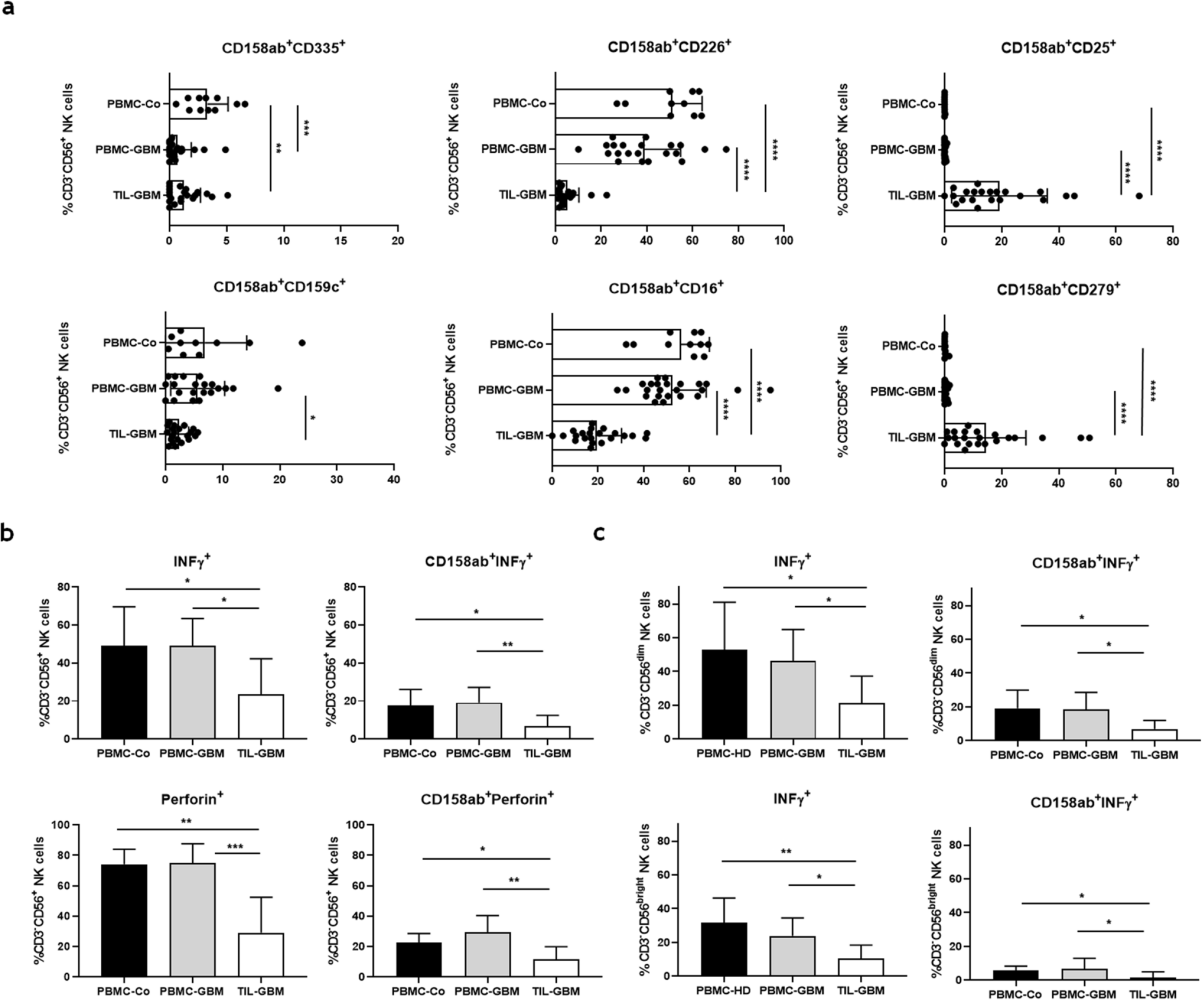
Expression profile and functional activity of CD158ab/KIR2DL-1,-2/3^+^ NK-cells. (**a**) Expression profile of selected NK-cell receptors and activation markers on CD158ab^+^ CD3^-^CD56^+^ NK-cells. The frequencies of CD158ab^+^ CD3^-^CD56^+^ NK-cells co-expressing the natural cytotoxic receptor CD335/NKp46, the activating NK receptors CD226/DNAM-1 and CD159c/NKG2C, CD16/Fc γ RIII, CD25/IL2Rα and CD279/PD-1 in peripheral blood and tumor cell suspensions of GBM patients and control individuals were determined. (**b**) IFN-γ and perforin expression of CD3^-^CD56^+^ NK-cells and CD158ab^+^ NK-cell subsets (n = 10): The proportion of IFN-γ and perforin producing cells within the entire CD3^-^CD56^+^ NK-cell population and the CD158ab^+^ NK-cell subsets of peripheral blood and tumor cell suspensions of GBM patients and control individuals after stimulation with PMA/Ionomycin were determined. (**c**) IFN-γ expression of CD56^dim^ and CD56^bright^ as well as CD158ab^+^ CD56^dim^ and CD56^bright^ NK-cell subsets (n = 10): The proportion of IFN-γ cells within the CD56^dim^ and CD56^bright^ NK-cell population and CD158ab^+^ CD56^dim^ and CD56^bright^ NK-cell subsets of peripheral blood and tumor cell suspensions of GBM patients and control individuals after stimulation with PMA/Ionomycin were determined. Mean values + /− SD are shown. P values were calculated using the Kruskal–Wallis test with post-hoc Dunn´s multiple comparison analysis (*p < 0.05,**p < 0.01, ***p < 0.001, ****p < 0.0001).

Subsequently, we analyzed the co-expression of selected activating receptors on CD158ab/ KIR2DL-1,-2/3^+^ CD3^-^CD56^+^ NK-cells. As shown in Fig. 2a, co-expression of CD226/DNAM-1 and CD159c/NKG2C was significantly lowered in tumor cell suspensions as compared to peripheral blood of GBM patients or control individuals. In contrast, CD335/NKp46 expression levels were reduced both in peripheral blood GBM patients and tumor cell suspensions. Moreover, the co-expression of CD16/Fcγ RIII was significantly reduced, whereas the co-expression of CD25/IL2Rα and CD279/PD-1 was significantly increased within the TIL population. Next, we evaluated the functional activity of CD158ab^+^ NK-cells after stimulation with PMA/Ionomycin. Both production of IFN-γ and perforin was significantly reduced in tumor-derived CD3^-^CD56^+^ and CD158 ab^+^ NK-cells after PMA/Ionomycin stimulation as compared to blood-derived NK-cells from GBM patients and control individuals (Fig. 2b). Further analysis revealed that the capacity to produce IFN-γ was significantly diminished both in tumor-derived CD56^dim^ and CD56^bright^ NK-cell subsets. Overall, we found higher frequencies of IFN-γ producing CD56^dim^ and CD158ab^+^ CD56^dim^ NK cells when compared to CD56^bright^ and CD158ab^+^ CD56^bright^ NK cells, respectively (Fig. 2c).

### Reversal of functional hypoactivity of GBM-derived NK-cells by IL-2 stimulation and blockade of inhibitory KIR2DL-1,-2/3

**Figure 3:**
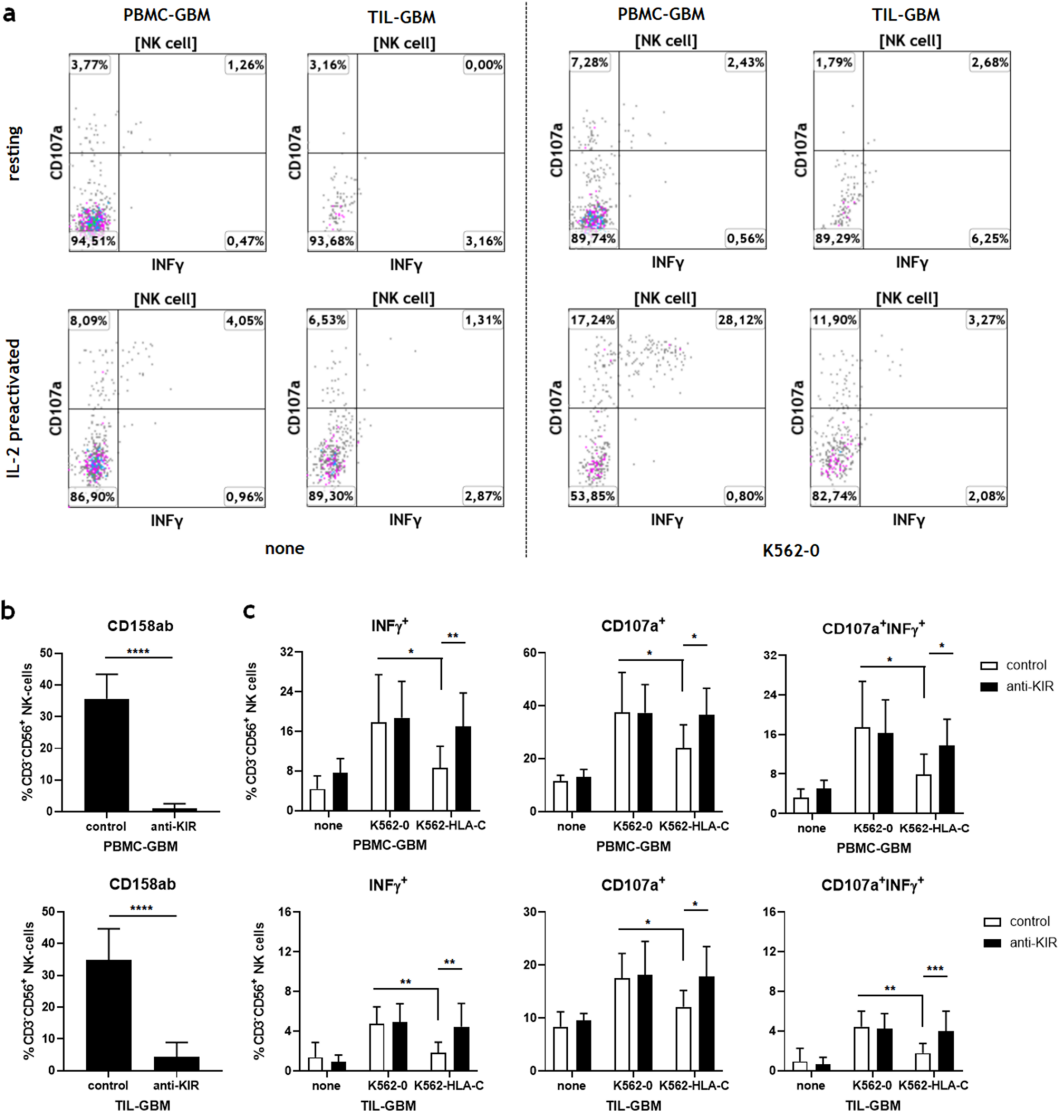
KIR2DL-1,-2/3 blockade reverses the functional hypoactivity of GBM-derived NK-cells. (**a**) Functional analysis of blood- and GBM-derived NK-cells: NK-cell responsiveness was assessed by cell surface mobilization of CD107a and IFN-γ production following K562 stimulation of resting or IL-2 preactivated PBMC and TIL at an effector to target ratio of 10:1 for 4.5 h. Representative dot plots are shown illustrating the capacity of blood and tumor-derived CD3^-^CD56^+^ NK-cells to express CD107a, IFN-γ or both CD107a and IFN-γ after stimulation with K562-0 cells or culture medium alone (none). (**b**,**c**) The activity and cytolytic potential of glioblastoma-derived NK-cells after KIR2DL-1,-2/3 blockade were assessed. (b) Blocking experiments revealed that Lirilumab significantly inhibited the binding of CD158a and CD158b mAb to blood- and tumor-derived NK-cells at a concentration of 30 μg/ml (****p < 0.0001). (**c**) IL-2 preactivated PBMC and TIL from different glioblastoma patients (n = 5) were pre-incubated with a human IgG4 isotype control mAb or Lirilumab (30 μg/ml), cocultured with culture medium (none), IFN-γ pretreated HLA-class I deficient K562 cells (K562-0) and HLA-C expressing K562 cells (K562-HLA-C) at an effector to target ratio of 10:1 for 4.5 h and then analyzed by flow cytometry. The frequencies of CD107a^+^, IFN-γ^+^and CD107a^+^IFN-γ^+^CD3^-^CD56^+^ NK-cells were determined. Mean values + /− SD are depicted. P values were calculated using the two-tailed Mann–Whitney test (*p < 0.05, **p < 0.01).

NK-cell responsiveness was further assessed by cell surface mobilization of CD107a indicating degranulation of cytolytic vesicles and IFN-γ production following K562 stimulation of resting and IL2-preactivated CD3^-^CD56^+^ NK-cells. As shown in Fig. 3a, both resting NK-cells from peripheral blood and tumor cell suspensions could not sufficiently be stimulated by K562 cells. IL-2 preactivation clearly enhanced the NK-cell responsiveness of blood-derived NK-cells, whereas tumor-derived NK-cells still showed reduced cytolytic activity and diminished IFN-γ production.

To test whether the blockade of CD158ab/KIRDL-1,-2/3 might facilitate the activation and cytotoxic potential of tumor-derived NK-cells, IL-2 preactivated TIL from GBM patients were preincubated with a human IgG4 isotype control mAb or Lirilumab (anti-KIR2DL-1,-2/3) and cocultured with IFN-γ pretreated HLA-C expressing K562 cells (K562-HLA-C) or HLA class-I deficient K562 cells (K562-0). Preceding blocking experiments revealed that Lirilumab significantly inhibited the binding of CD158a and CD158b monoclonal antibodies to blood- as well as tumor-derived NK-cells at a concentration of 30 μg/ml (Fig. 3b). As shown in Fig. 3c, the responsiveness of blood- and tumor-derived NK-cells was significantly reduced after stimulation with HLA-C expressing K562 cells when compared to HLA class-I deficient K562 cells. However, KIR2DL-1,-2/3 blockade by Lirilumab reversed NK cell inhibition and significantly enhanced the percentages of CD107a^+^, IFN-γ^+^ and CD107a^+^/IFN-γ^+^ CD3^-^CD56^+^ NK-cells in the presence of HLA-C expressing but not HLA class-I deficient K562 cells. Lirilumab alone did not influence the effector functions of IL-2 preactivated NK-cells.

**Figure 4:**
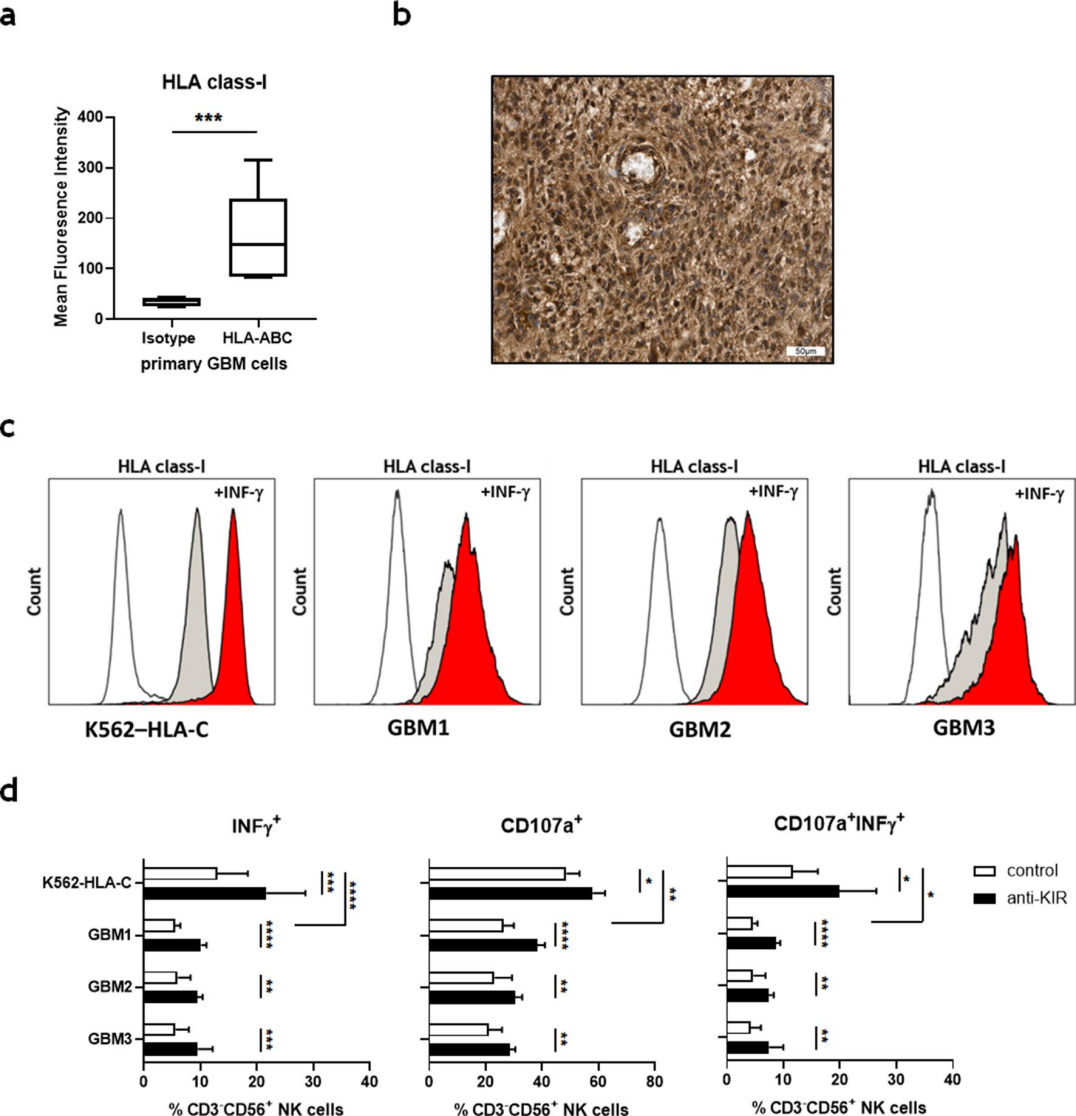
KIR2DL-1,-2/3 blockade improves the NK-cell stimulatory properties of HLA class-I expressing GBM cells. (**a**) Primary GBM cell cultures were prepared and evaluated for the expression of HLA class-I molecules (HLA-ABC) by flow cytometry (n = 5). (**b**) Representative immunohistochemical staining of HLA class I-expressing cells within glioblastoma tissue. (**c**) Expression of HLA class-I molecules on unstimulated (grey histograms) or IFN-γ pretreated (red histograms) K562-HLA-C and primary GBM cells (GBM1-3). (**d**) IL-2 preactivated PBMC from glioblastoma patients (n = 3) were pre-incubated with a human IgG4 isotype control mAb or Lirilumab (30 μg/ml), cocultured with IFN-γ pretreated HLA-C expressing K562 cells (K562-HLA-C) or primary GBM cells at an effector to target ratio of 10:1 for 4.5 h and then analyzed by flow cytometry. The frequencies of CD107a^+^, IFN-γ^+^ and CD107a^+^IFN-γ^+^ CD3^-^CD56^+^ NK-cells were determined. Mean values + /− SD are depicted. P values were calculated using the two-tailed Mann–Whitney test (*p < 0.05, **p < 0.01, ***p < 0.001, ****p < 0.0001).

To further prove the relevance of KIR blockade in GBM, primary cell cultures from tumor material were prepared and tested for HLA class-I expression. Flow cytometry and immunohistochemistry confirmed the expression of significant levels of HLA class-I molecules (Fig. 4a,b). INF-γ pretreatment of GBM cells and K562-HLA-C cells further increased HLA class-I expression (Fig. 4c). Subsequently, GBM cells (GBM1-3) were tested for their capacity to stimulate IL-2 preactivated NK cells obtained from peripheral blood of GBM patients in the absence or presence of Lirilumab. As shown in Fig. 4d, blockade of KIR2DL-1, 2/3 by Lirilumab significantly enhanced the NK cell responsiveness with increased expression of CD107a^+^, IFN-γ^+^ and CD107a^+^/IFN-γ^+^ but GBM cells were less potent stimulators of NK cells than K562-HLA-C cells.

### Enhanced IL-2 production of GBM-derived memory T-cells after combined anti-CD3/anti-CD28 stimulation

**Figure 5:**
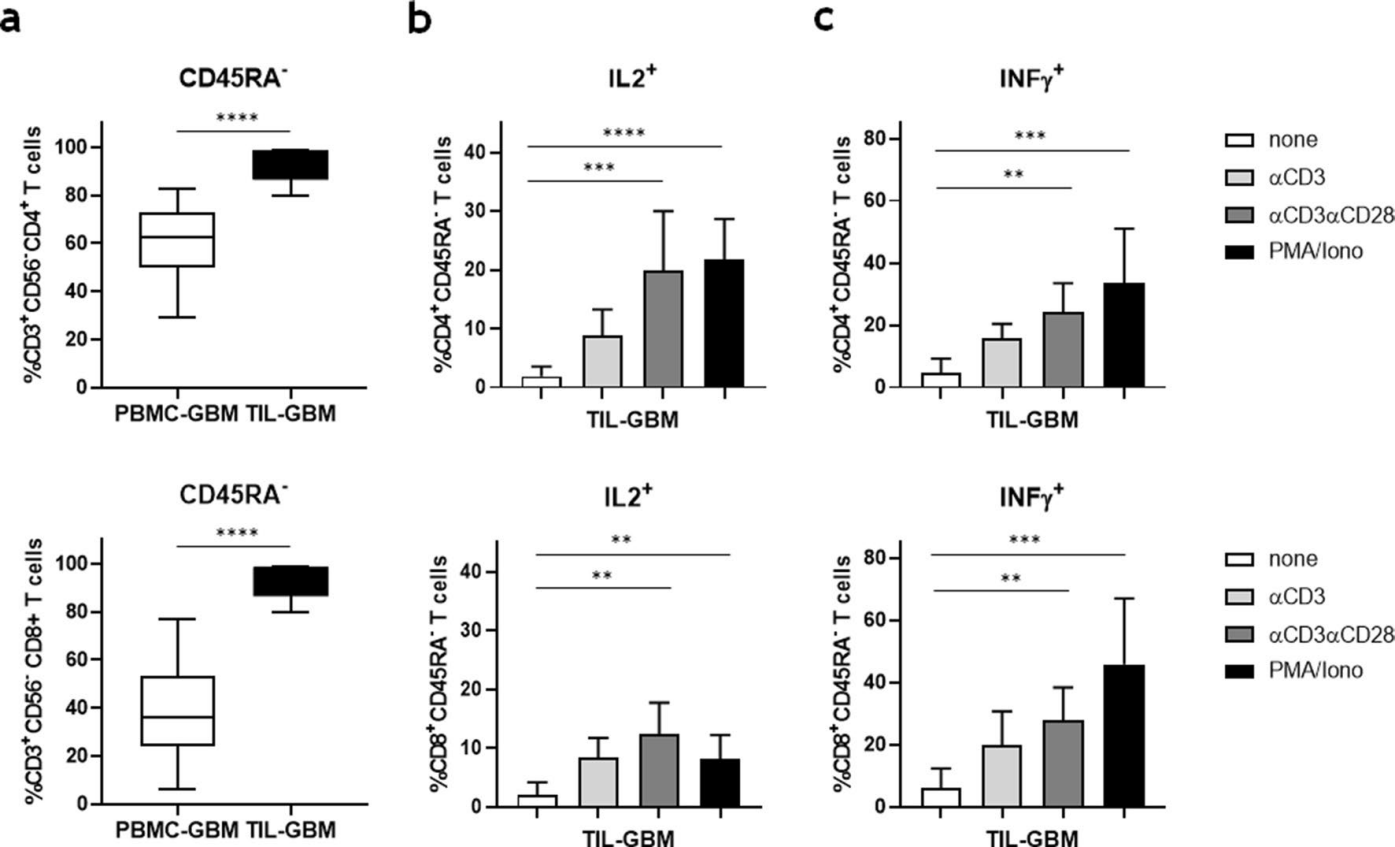
Functional analysis of GBM-derived CD4^+^ and CD8^+^ memory T-cells. (**a**) The frequency of CD4^+^ and CD8^+^ CD45RA^-^ memory T-cells in PBMC and TIL of GBM patients were determined by flow cytometry (n = 10). Mean values + /− SD are depicted. P values were calculated using the two-tailed Mann–Whitney test (****p < 0.0001). (**b**) IL-2 and (**c**) IFN-γ production of CD4^+^ and CD8^+^ CD45RA^-^ memory T-cells after stimulation with culture medium (none), immobilized anti-CD3 mAb, immobilized anti-CD3/anti-CD28 mAb or PMA/Ionomycin for 6 h in the presence of Brefeldin A and Monsenin (n = 10). Mean values + /− SD from five different GBM patients are depicted. P values were calculated using the Kruskal–Wallis test with post-hoc Dunn´s multiple comparison analysis (**p < 0.01, ***p < 0.001, ****p < 0.0001).

As IL-2 preactivation is critical for the cytolytic capacity of NK-cells in glioblastomas, we further evaluated whether tumor-infiltrating T-cells were able to produce IL-2 upon stimulation. T-cell subset analysis of tumor cell suspensions revealed that glioblastomas are mainly infiltrated by CD4^+^ and CD8^+^ CD45RA^-^ memory T-cells (Fig. 5a). Both cell populations, with a predominance of CD4^+^ memory T-cells, showed enhanced IL-2 production after stimulation with immobilized anti-CD3 or anti-CD3/anti-CD28 mAbs. However, combined TCR and costimulatory molecule signalling was necessary to produce similar amounts of IL-2 compared to T-cells stimulated with PMA/Ionomycin (Fig. 5b). Likewise, significant amounts of IFN-γ were produced by CD4^+^ and CD8^+^ CD45RA^-^ memory T-cells upon stimulation with anti-CD3 and anti-CD28 mAbs (Fig. 5c).

## Discussion

Alterations in NK-cell subset distribution and NK-cell characteristics have been detected in numerous cancers^18^. Here we report scarce infiltration of glioblastomas by NK-cells that are functionally impaired as indicated by diminished expression of NCRs and co-receptors, downregulation of CD16, reduced degranulation and IFN-γ production, and upregulation of CD25 and PD-1.

Using multicolor flow cytometry, we demonstrated unaltered numbers and distribution of NK subsets in the peripheral blood of glioblastoma patients as compared to control individuals. To the contrary, we identified only a minor fraction of CD3^-^CD56^+^ NK-cells within the CD45^+^ leucocyte population at the tumor site suggesting that NK-cell entry is impaired in glioblastomas. Therefore, strategies to increase NK cell infiltration into glioblastomas are mandatory to enhance antitumor efficacy^19,20^.

Moreover, we observed an increased fraction of NK-cells with preferential expression of CD158ab/KIR2DL-1,-2/3, and downregulation of CD159a/NKG2A within the tumor tissue. Since the KIR repertoire of NK-cells is largely influenced by self HLA class-I molecules during NK-cell maturation, the appearance of tumor-infiltrating NK-cells expressing KIR receptors might be explained by the fact that glioblastomas usually express high levels of HLA class-I molecules^21^. Indeed, immunohistochemistry of primary tumor material and flow cytometric analysis of primary GBM cell lines demonstrated prominent expression of HLA class-I molecules in all cases investigated. Higher expression of CD158a and CD158b KIR receptors on tumor-infiltrating NK-cells that correlated with diminished NK-cell cytotoxicity and tumor progression has also been observed in patients with other solid tumors such as melanoma or non-small cell lung cancer^22–24^. We could, however, not detect any significant association between NK-cell numbers, KIR expression and clinical parameters in our study.

Both downregulation of activating receptors and shedding of corresponding NK ligands were identified as relevant mechanisms used by tumor cells to evade NK-cell directed antitumor immunity^25–29^. Similarly, our analysis revealed significantly reduced expression of NCRs like CD335/NKp46 and activating co-receptors such as CD226/DNAM-1 on tumor-derived CD158ab^+^ NK-cells accompanied by a restricted capacity to produce IFN-γ or perforin after stimulation with PMA/Ionomycin. We also found a significant upregulation of CD25 and PD-1 on CD158ab^+^ NK-cells consistent with an exhausted phenotype which has also been described for other tumor entities or tumor infiltrating cell types^30,31^. In accordance with the reduced expression of CD159a/NKG2A, we detected a significant downregulation of CD159c/NKG2C on CD158ab^+^ NK-cells from tumor stroma. Although we did not include the analysis of CD314/NKG2D in our study, previous reports indicate that its expression on NK-cells in glioblastoma patients is equally reduced. Importantly, we also found that CD16/Fc γ RIII was markedly decreased on tumor-derived CD158ab^+^ NK-cells which further underlines that NK-cells from glioblastoma tissue are highly immunocompromised. Moreover, we previously demonstrated that the expression of CD16/Fc γ RIII was also downregulated on tumor-associated myeloid cells in GBM^31^. Therefore, triggering NK-cell mediated antibody-dependent cell-mediated cytotoxicity (ADCC) through crosslinking therapeutic antibodies to CD16 does not seem to be a promising strategy for GBM^32^. We note that a minor fraction (about 1–2%) of CD56^+^ monocytes/macrophages could be a confounding factor in interpreting our data.

It is well known that a certain threshold must be surpassed by the sum of activating and competing inhibitory signals to prompt NK cytotoxicity and cytokine release^33^. Our experiments revealed that at least two signals were necessary to overcome unresponsiveness of tumor-derived NK-cells in GBM: Firstly, tumor-derived NK need to be preactivated by IL-2 stimulation. Secondly, blocking of the inhibitory receptor KIR is a prerequisite to enable proper NK-cell activation by NK ligands on HLA-class I-expressing target cells which delivered strong inhibitory signals to tumor-derived NK cells. This could be even more relevant in case of an inflammatory tumor microenvironment with secretion of IFN-γ and further upregulation of HLA-class I molecules on GBM cells. In comparison to NK-cell sensitive K562 cells, GBM cells showed a diminished capacity to activate NK cells. Extended analysis revealed that GBM cells expressed higher levels of other inhibitory NK ligands such as non-classical HLA-E molecules, CD155 and MICA/B when compared to K562 cells (Suppl. Fig. 1), indicating that additional inhibition of these molecules is required to elicit even stronger NK-cell-mediated immunity^34–36^.

As triggering of freshly isolated tumor-derived NK-cells in glioblastomas is substantially impaired, we also postulated that IL-2 signaling is required to upregulate activating receptors on tumor-derived NK-cells in glioblastomas as has been previously reported for other tumor entities^37–39^. Indeed, additional experiments verified that IL-2 increased the expression of CD335/NKp46, CD336/NKp44, CD337/NKp30, CD314/NKG2D and CD159c/NKG2C on GBM-derived CD158ab^+^ NK-cells (Suppl. Fig. 4).

The use of exogenous IL-2 to enhance NK-cell activity has been evaluated in glioblastoma and other cancer patients but was limited by pronounced in vivo toxicity^40,41^. Moreover, IL-2 is known to control and boost the growth of regulatory T-cells within the tumor microenvironment thus impeding its own anti-tumor activity^42–44^. Therefore, new selective IL-2 therapeutics with preferential activation of effector T-cells are under development and currently tested in clinical trials, including pegylated IL-2 agents, IL-2 protein mimetics or partial IL-2 agonists. Considering that PD-1 is highly upregulated on tumor-derived NK-cells (Fig. 3B) and effector T-cells^31^, IL-2 antibody fusion proteins, such as IL-2variant-anti-PD-1 mAb fusion proteins, might also be attractive candidates to enhance T- and NK-cell mediated anti-tumor immunity in GBM^45^.

Alternative ways for NK-cell activation and upregulation of NCRs comprise the stimulation of endogenous IL-2 production within the tumor microenvironment. Here, we have shown that CD4^+^ and CD8^+^ memory effector T-cells in GBM can be stimulated to produce IL-2 using immobilized antibodies specific for the TCR-CD3 complex and the costimulatory molecule CD28. Additional analysis revealed a strong upregulation of activation markers such as CD69 and CD337/NKp30 on CD158ab^+^ NK-cells after combined anti-CD3/anti-CD28 treatment of GBM-derived T-cells. Blocking experiments with a neutralizing anti-CD25 monoclonal antibody confirmed that this effect was IL-2 dependent (Suppl. Fig. 5).

Several other agonistic antibodies targeting costimulatory molecules, such as the TNF receptor superfamily members (TNFRSF) CD137 and OX40, that are expressed on tumor-infiltrating T-cells, are currently under investigation in cancer immunotherapy^46,47^. Since TNFRSF agonist antibodies require crosslinking via Fc γ R and the availability of Fc γ R-expressing cells in GBM, as shown here, is limited, new approaches using dual agonist bispecific antibody targeting CD137 and OX40 in an Fc γ R-independent way, might be of high potential in combination with KIR blockade in the treatment of GBM^48^.

In summary, our data demonstrate that inhibitory CD158ab/KIR2DL-1,-2/3 are preferentially expressed on glioblastoma-derived NK-cells and that KIR blockade is a promising tool to enhance NK-cell activity against GBM, but additional activating signals must be delivered to break functional hypoactivity of NK-cells present within the tumor.

## Material and methods

### Preparation of blood and tumor samples

Blood and freshly resected tumor material were obtained from patients who had been diagnosed with GBM according to the existing WHO classification (primary GBM, n = 50; GBM at first recurrence, n = 10). Tumor material was mainly obtained by ultrasonic aspiration using the CUSA Excel® system (Integra Radionics Inc., MA, USA)^49^. Tumor fragments were washed extensively to discard blood and suction fluid. Tumor infiltrating leucocytes (TIL) and peripheral blood mononuclear cells (PBMC) were isolated as previously described^31^. In addition, we analyzed blood samples from age and sex-matched control individuals (n = 32) (for details see Table 1).

**Table 1:** Patient characteristics.

Numbers	Patients = 60	Controls = 32
**Diagnosis**		
primary GBM IDH wildtype (n)	50 (83%)	
recurrent GBM IDH wildtype (n)	10 (17%)	
**Sex**		
male (n)	34 (57%)	18 (56%)
female (n)	26 (43%)	14 (44%)
**Median age**		
years (range)	61 (41–83)	61 (41–81)
**Resection status**		
complete	35 (58%)	
incomplete	25 (42%)	
**Steroids**		
received	38 (63%)	
not received	22 (37%)	
**MGMT status**		
methylated	26 (43%)	
unmethylated	34 (57%)	

### Cells

NK-sensitive human erythroid leukemia K562 cells (ATCC, Cat# CCL-243, RRID: CVCL_0004) stably transfected with HLA-Cw3 and HLA-Cw4 (K562-HLA-C) were kindly provided by Prof. Irma Joosten, Radboud UMC, Dept. of Immunology, Nijmegen, Netherlands. HLA class I-deficient K562 cells transfected with empty vectors were used as control cells (K562-0)^8^. Cells were cultured in complete medium (IMDM supplemented with 10% (vol/vol) heat-inactivated FCS, 1% (vol/vol) sodium pyruvate and 1% (vol/vol) Pen/Strep (all from Gibco/ThermoFisher, MA, USA). Primary GBM cell cultures were prepared by adding CUSA aspirates to 25 cm^2^ cell culture flasks in complete medium. Early passages were analyzed by flow cytometry. Primary GBM cells from one donor (GBM1), U-251MG cells (GBM2, ECACC Cat# 09,063,001, RRID:CVCL_0021), and previously described HTZ-349 cells (GBM3)^50^ were used for functional assays. All cells were stained for HLA class-I (Anti-HLA-ABC), CD155/PVR, MICA/B (MHC class I chain-related protein A and B) and HLA-E molecules (all Biolegend, UK, 1:100) (Suppl. Fig. 1).

### Multicolor flow cytometry

Freshly prepared TIL and PBMC were washed, filtered through a 70 μM cell strainer (CellSystems, Germany), and immediately stained with a panel of directly labeled mAbs (for details see Supplementary Methods). The viability of isolated cells was generally > 95% as evaluated by Trypan Blue staining. For labeling of cell surface molecules, 2–5 × 10^5^ cells were stained with fluorochrome-conjugated antibodies diluted in PBS/0.5% BSA/2 mM EDTA and incubated for 30 min at 4 °C. IL-2 blocking experiments were performed by adding purified anti-CD25 mAbs (Ebiosience, clone B-B10, 50 µg/ml) to the cultures. For intracellular detection of IFN-γ and perforin, TIL and PBMC were stimulated with phorbol 12-myristate 13-acetate (PMA, Sigma-Aldrich, Germany, 100 ng/mL) plus ionomycin (Sigma-Aldrich; 1 μg/mL) for 4.5 h in the presence of Brefeldin A (ThermoFisher, 3 μg/ml). Cells were then stained with NK-cell antibodies, further processed with the Fixation/Permeabilization Kit (ThermoFisher) and labeled with anti-perforin-PB and APC-conjugated IFN-γ mAb (Biolegend) diluted in permeabilization solution according to the manufacturer’s instructions. All samples were analyzed using the Navios™ flow cytometer and the Kaluza 2.1 Software (Beckman Coulter). The gating strategy for NK-cell subsets is illustrated in Suppl. Fig. 2.

### Immunohistochemistry

Immunohistochemical staining of formalin-fixed and paraffin-embedded tumor tissue for KIR2DL-1/2/3 (1:500, rabbit monoclonal [EPR22541-47], #ab224697 Abcam, Cambridge, MA) and HLA-ABC (1:100, rabbit polyclonal, #PA5-104,359, ThermoFisher) was performed using the streptavidin–biotin peroxidase technique on an automated staining system (Link48, DAKO). The slides were counterstained using hematoxylin.

### Functional analysis (CD107a mobilization/IFN– γ release assay)

To test the functional capacity of NK-cells, freshly isolated TIL and PBMC were left untreated or preactivated with recombinant IL-2 (100 IU/mL, Peprotech, Germany) for 72 h in complete medium. Cells were harvested and the proportion of viable NK-cells within the PBMC and TIL fraction was determined by flow cytometry. Cells were transferred to 96-well round bottom microplates (Corning^TM^Costar™) in complete medium in duplicates, and IFN-γ pretreated (100 IU/ml, 48 h) HLA-C expressing K562 cells (K562-HLA-C), HLA class-I deficient K562 cells (K562-0) or primary GBM cells were added at an effector to target ratio of 10:1. Cells were mixed by gentle pipetting, spun down at 300 g for 2 min and cocultured for 4.5 h at 37 °C in the presence of 2 µM Monensin, 3 μg/ml Brefeldin A (ThermoFisher) and an APC-conjugated anti-CD107a mAb (Biolegend). After incubation, cells were washed, stained for extracellular NK-cell markers and intracellular IFN-γ, and analyzed by flow cytometry. For blocking experiments, we used the fully human IgG4 anti-KIR2DL-1,-2/3 mAb (Lirilumab/IPH2102/BMS-986015) and a purified human IgG4 isotype control mAb (Biolegend). Effector cells were pre-incubated with Lirilumab or control mAb at a concentration of 30 µg/ml and target cells were added without washing after 30 min (37 °C, 95% humidity, 5% CO2). Samples were further processed as described above.

### T-cell stimulation assay

96-well flat bottom microplates (Corning™ Costar™) were precoated with anti-CD3 or anti-CD3/anti-CD28 mAbs (10 μg/ml each, diluted in PBS, 100 μl/well) for 24 h at 4 °C (Biolegend). Without washing, freshly isolated TIL (2–5 × 10^5^ cells/well) were transferred to the plates in complete medium in duplicates. Cells were spun down at 300 g for 2 min and cocultured for 6 h at 37 °C in the presence of 2 µM Monensin and 3 µg/ml Brefeldin A. Subsequently, cells were stained for extracellular T-cell markers, followed by intracellular labeling using FITC-conjugated anti-IL2 mAb or PE-conjugated anti-IFN-γ mAb (Biolegend) according to the manufacturer’s instructions (ThermoFisher), and analyzed by flow cytometry. The gating strategy for T-cell subsets is illustrated in Suppl. Fig. 3.

### Statistics

The Kolmogorov–Smirnov procedure was performed to exclude normal distribution of the data, followed by the nonparametric two-tailed Mann–Whitney test to examine significant differences between 2 groups, and the Kruskal–Wallis test with post hoc Dunn multiple comparison analysis to test for significant differences between 3 groups. Values of *p* < 0.05 were considered significant. Error bars represent standard deviations (SD). GraphPad Prism 8.4.2 (GraphPad Software Inc., CA, USA) was used for statistical analyses.

### Ethics declarations

The studies involving human subjects were carried out in accordance with the principles of the 1964 Declaration of Helsinki and its later amendments, and were approved by the ethics committee of the University of Muenster Medical School (file numbers 2010–262-f-S, 2010–461-f-S, and 2019–276-f-S). All patients/participants provided their written informed consent to participate in this study.

## Supplementary Information

Supplementary Information.

## Data Availability

All data generated or analysed during this study are included in the article/supplementary material. Data are available from the corresponding author on reasonable request.
