# Supplementary material for: Blockade of inhibitory killer cell immunoglobulin-like receptors and IL-2 triggering reverses the functional hypoactivity of tumor-derived NK-cells in glioblastomas
Source: Sci Rep. 2022 Apr 26;12:6769. doi: 10.1038/s41598-022-10680-4 (PMC9042843; doi:10.1038/s41598-022-10680-4)
Supplement: Supplementary file 1 — Supplementary Information. [file 41598_2022_10680_MOESM1_ESM.pdf]

SUPPLEMENTARY MATERIAL:

Suppl.Fig.1

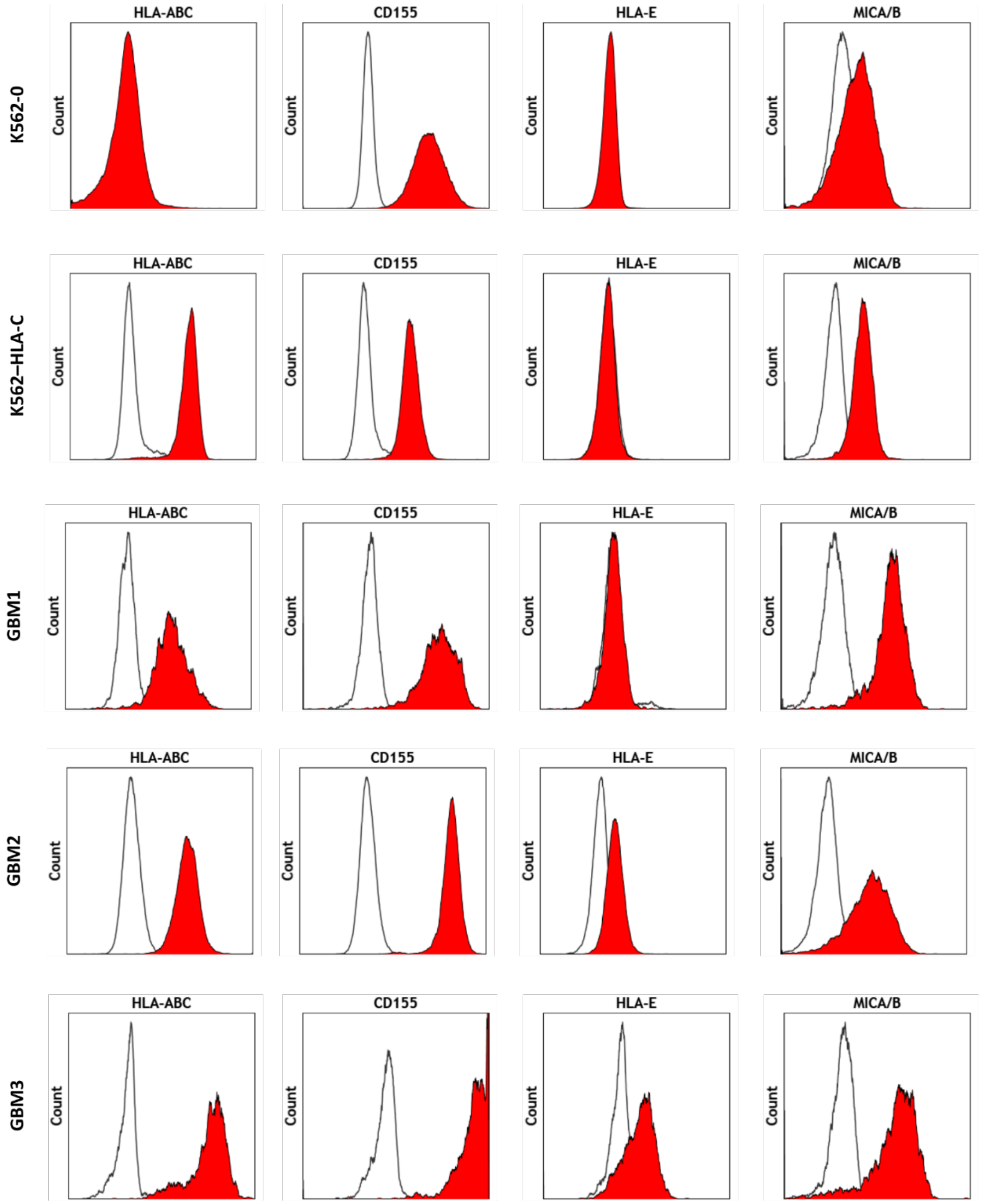

Suppl.Fig.2

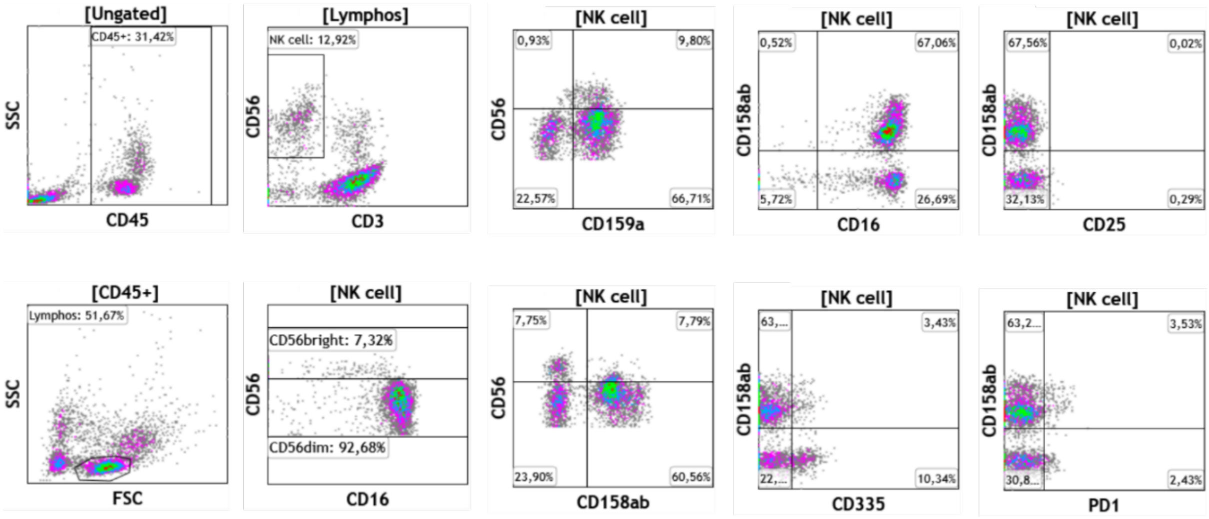
[Ungated]
CD45+: 31,42%
SSC
CD45
[Lymphos]
NK cell: 12,92%
CD56
CD3
[NK cell]
0,93%
9,80%
22,57%
66,71%
CD56
CD159a
[NK cell]
0,52%
67,06%
5,72%
26,69%
CD158ab
CD16
[NK cell]
67,56%
0,02%
32,13%
0,29%
CD158ab
CD25
[CD45+]
Lymphos: 51,67%
SSC
FSC
[NK cell]
CD56bright: 7,32%
CD56dim: 92,68%
CD56
CD16
[NK cell]
7,75%
7,79%
23,90%
60,56%
CD56
CD158ab
[NK cell]
63,...
3,43%
22,...
10,34%
CD158ab
CD335
[NK cell]
63,2...
3,53%
30,8...
2,43%
CD158ab
PD1

Suppl.Fig.3

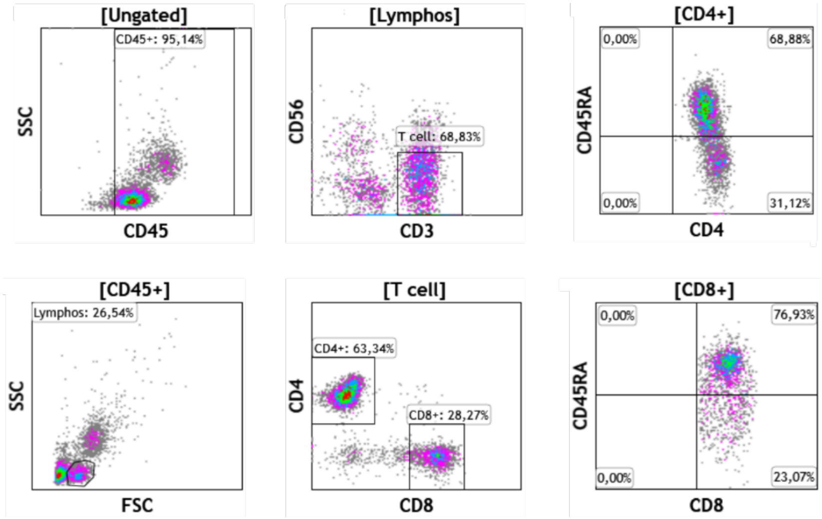

[Ungated]
CD45+: 95,14%
SSC
CD45
[Lymphos]
T cell: 68,83%
CD56
CD3
[CD4+]
0,00%
68,88%
0,00%
31,12%
CD45RA
CD4
[CD45+]
Lymphos: 26,54%
SSC
FSC
[T cell]
CD4+: 63,34%
CD8+: 28,27%
CD4
CD8
[CD8+]
0,00%
76,93%
0,00%
23,07%
CD45RA
CD8

Suppl.Fig.4

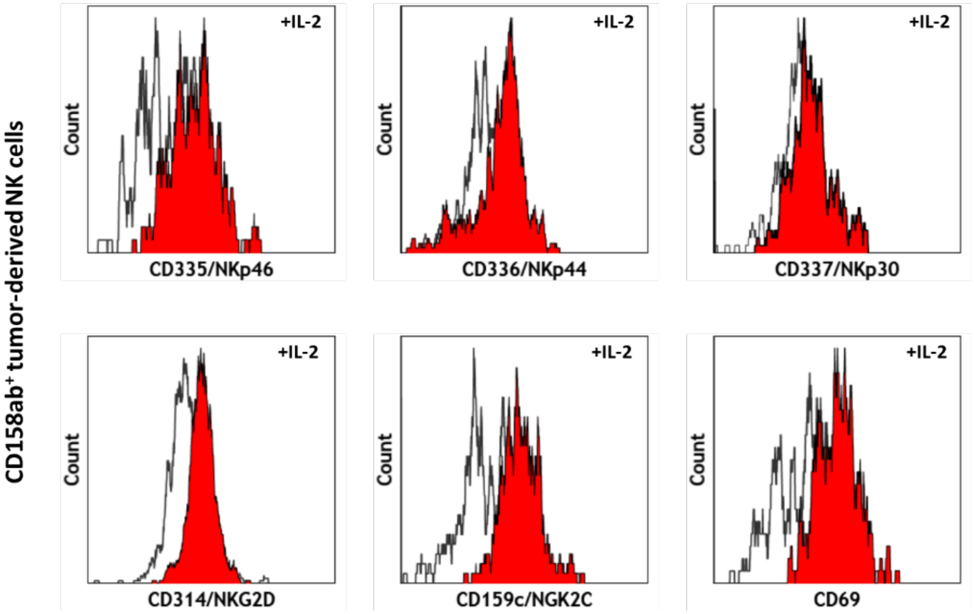
CD158ab⁺ tumor-derived NK cells
+IL-2
Count
CD335/NKp46
+IL-2
Count
CD336/NKp44
+IL-2
Count
CD337/NKp30
+IL-2
Count
CD314/NKG2D
+IL-2
Count
CD159c/NGK2C
+IL-2
Count
CD69

Suppl.Fig.5

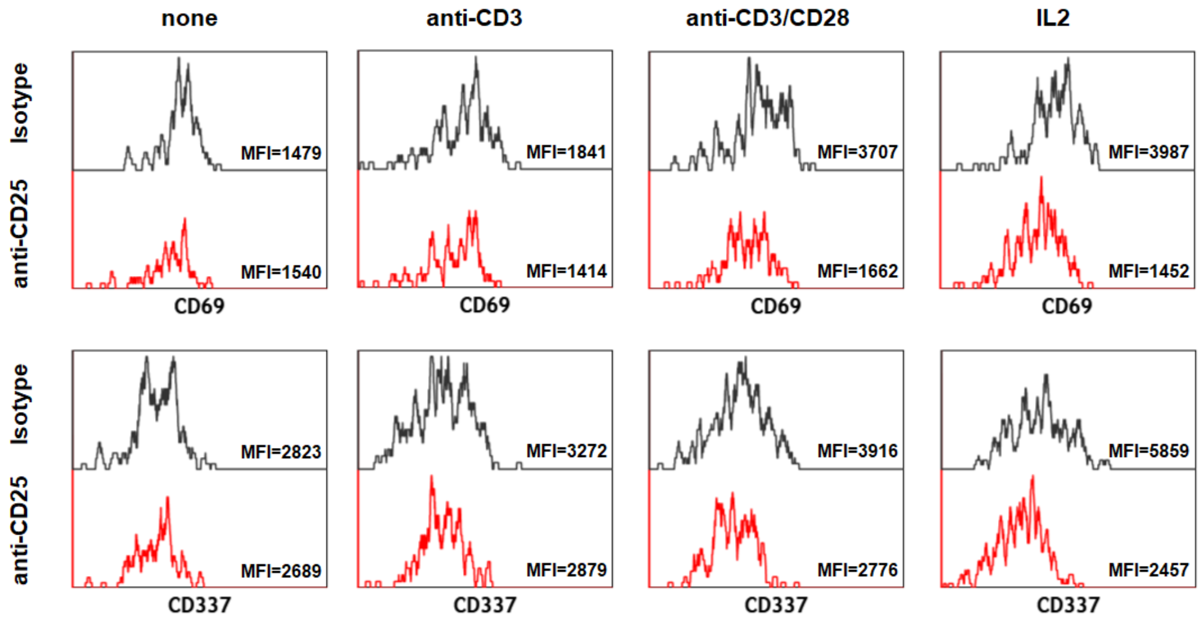

none
anti-CD3
anti-CD3/CD28
IL2
Isotype
anti-CD25
MFI=1479
MFI=1540
MFI=1841
MFI=1414
MFI=3707
MFI=1662
MFI=3987
MFI=1452
CD69
Isotype
anti-CD25
MFI=2823
MFI=2689
MFI=3272
MFI=2879
MFI=3916
MFI=2776
MFI=5859
MFI=2457
CD337

Suppl.Fig. 6

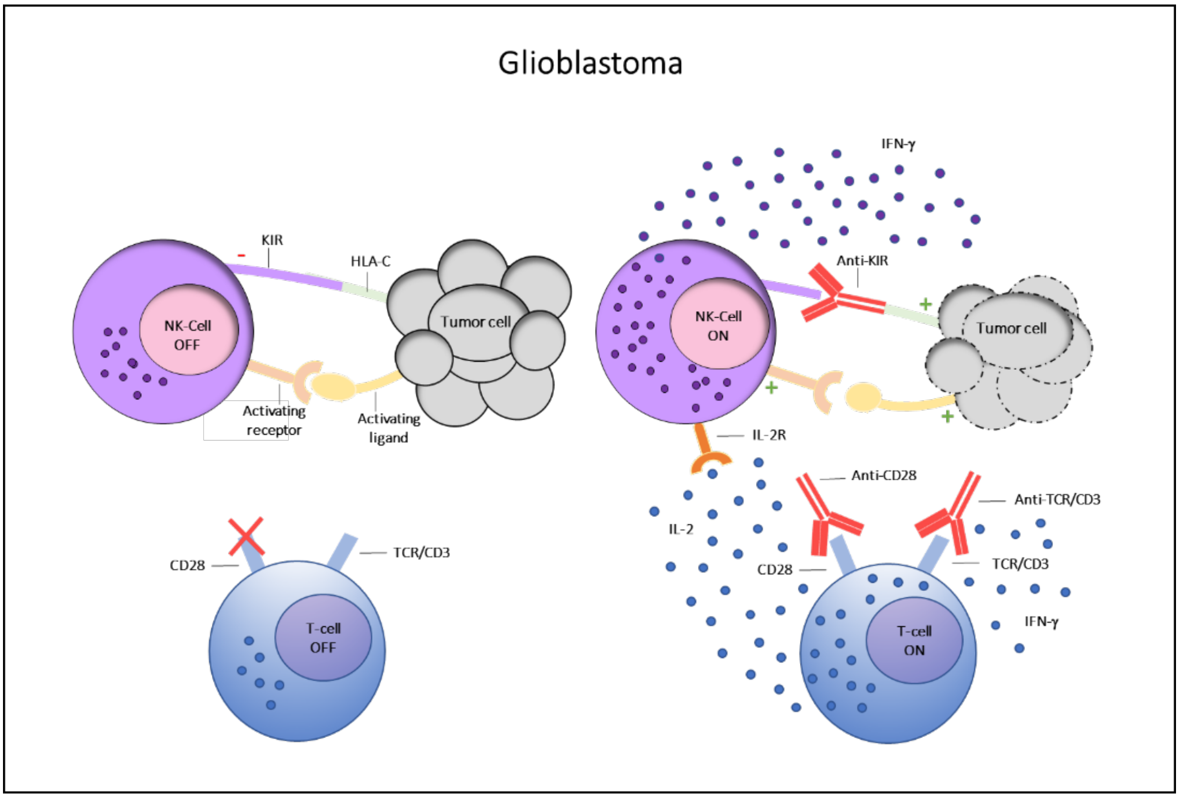
Glioblastoma
IFN-γ
KIR
HLA-C
NK-Cell
OFF
Tumor cell
Activating
receptor
Activating
ligand
Anti-KIR
NK-Cell
ON
Tumor cell
IL-2R
Anti-CD28
Anti-TCR/CD3
IL-2
CD28
TCR/CD3
T-cell
ON
IFN-γ
CD28
TCR/CD3
T-cell
OFF

SUPPLEMENTARY FIGURE LEGENDS:

Suppl.Fig.1: Expression of selected NK ligands on HLA-class I deficient K562 cells (K562-0), HLA-C expressing K562 cells (K562-HLA-C) and primary GBM cells (GBM1-3). Cells were stained with anti-HLA-ABC-FITC (W6/32), anti-CD155-PE, anti-HLA-E-APC and anti-MICA/B-PC7 mAbs (red histograms) or corresponding isotype controls (black lines). All mAbs were obtained from Biolegends, UK.

Suppl.Fig.2: Gating strategy for NK-cell subsets. To determine the frequency and phenotype of different NK subsets, $CD45^+$ leukocytes were selected in a CD45 versus forward scatter channel (FSC) plot. $CD45^+$ cells were then displayed in a forward (FSC) versus sideward scatter channel (SSC) plot to identify lymphocytes. To distinguish between $CD3^-CD56^+$ NK-cells and $CD3^+CD56^-$ T-cells, lymphocytes were displayed in a CD3 versus CD56 plot. $CD3^-CD56^+$ NK-cells were further split into $CD56^{bright}$ and $CD56^{dim}$ cells. CD3-CD56+ NK-cells were also analyzed for the expression of CD159a/NKG2A, CD158ab/KIR2DL-1,-2/3 and the co-expression of CD158ab and CD16/FcγRIII, CD335/NKp46, CD25/IL-2Rα or CD279/PD-1 (selected markers).

Suppl.Fig.3: Gating strategies for T-cell subsets. To determine the frequency of different T cell subsets, $CD45^+$ leukocytes were selected in a CD45 versus forward scatter channel (FSC) plot. $CD45^+$ cells were then displayed in a forward (FSC) versus sideward scatter channel (SSC) plot to identify lymphocytes. Lymphocytes were displayed in a CD3 versus CD56 plot to identify $CD3^+CD56^-$ T cells. T-cells were then split into $CD4^+$ and $CD8^+$ T-cells and further divided in $CD45RA^+$ naïve or $CD45RA^-$ memory T-cells.

Suppl.Fig.4: Expression of activating NK receptors upon IL-2 stimulation. Upregulation of activating NK receptors after IL-2 preactivation of $CD158ab/KIR2DL\text{-}1,\text{-}2/3^+$ NK cells obtained from glioblastoma tissue: Co-expression of the activating NK cell receptors CD335/NKp46, CD336/NKp44, CD337/NKp30, CD314/NKG2D, CD159c/NKG2C and the activation marker CD69 on $CD158ab^+$ $CD3^-CD56^+$ NK cells were determined by flow cytometry directly after preparation of the tumor cell suspensions (black lines) or after preincubation with IL-2 for 3 days (red histograms).

Suppl.Fig.5: Representative histogram overlay illustrating the expression levels of CD69 and CD337/NKp30 on CD158ab^{+} CD3^{-}CD56^{+} NK-cells after stimulation with culture medium (none), immobilized anti-CD3 mAb or immobilized anti-CD3/anti-CD28 mAb in the presence of human IgG1 isotype control mAb (black lines) or anti-CD25 mAb (red lines) for 48h.

Suppl.Fig.6: Graphical abstract

SUPPLEMENTARY METHODS:

Multicolor flowcytometry:

The mAbs (obtained from Beckman Coulter, Germany, and Biolegend, 1:100) used for staining included anti-CD158a/KIR2DL-1-FITC and anti-CD158b/KIR2DL-2/3-FITC, anti-CD159a/NKG2A-PE or anti-CD159c/NKG2C-PE, anti-CD3-ECD, anti-CD16-ECD or anti-CD56-ECD, anti-CD56-PC5.5 or anti-CD335/NKp46-PC5.5, anti-CD56-PC7, anti-CD159a/NKG2A-PC7 or anti-CD337/NKp30-PC7, anti-CD336/NKp44-APC or anti-CD226/DNAM-1-APC, anti-CD25/IL2Rα-APC-A700 or anti-CD69-A700, anti-CD279/PD1-APC-A750 or anti-CD3-APC-A750, anti-CD16-PB, anti-CD45-KrO or anti-CD3BV510. For analysis of T cell subpopulations (naïve vs memory T cells), we used the following mAbs: anti-CD4-ECD, anti-CD197-PC5.5, anti-CD45RA-PC7, anti-CD56-APC, anti-CD69-A700, anti-CD3-APC-A750, anti-CD8-PB, anti-CD45-KrO.
